# Differential expression patterns of Nqo1, AKR1B8 and Ho-1 in the liver and small intestine of C57BL/6 mice treated with sulforaphane

**DOI:** 10.1016/j.dib.2015.09.029

**Published:** 2015-10-03

**Authors:** Lin Luo, Yeru Chen, Deqi Wu, Jiafeng Shou, Shengcun Wang, Jie Ye, Xiuwen Tang, Xiu Jun Wang

**Affiliations:** a Department of Pharmacology, School of Medicine, Zhejiang University, Hangzhou 310058, PR China; b Department of Biochemistry and Genetics, School of Medicine, Zhejiang University, Hangzhou 310058, PR China; c Department of Pharmacology, University of Nantong, Nantong, PR China

**Keywords:** Nrf2, Sulforaphane, NQO1, AKR1B8, HO-1

## Abstract

This data article contains complementary figures and results related to the research article entitled “butylated hydroxyanisole induces distinct expression patterns of Nrf2 and detoxification enzymes in the liver and small intestine of C57BL/6 mice” (Luo et al., 2015 [1]), which defined the basal and butylated hydroxyanisole (BHA)-induced expression patterns of Phase II enzymes Nqo1, AKR1B8, and Ho-1 in the liver and small intestine of C57BL/6 mice. Sulforaphane [1-isothiocyanato-4-(methylsulfinyl)butane] (SFN), a naturally occurring isothiocyanate derived from cruciferous vegetables, is a highly potent inducer of phase II cytoprotective enzymes. This dataset reports the histological changes of Nqo1, AKR1B8, and Ho-1 in wild-type (WT) and *Nrf2*^*-/-*^ mice induced by SFN. The mice were given a 25 mg/kg single oral dose of SFN for 24 h and 48 h. Immunohistochemistry revealed that, in the liver from WT mice, SFN increased Nqo1 staining in hepatocytes with slight higher staining in the pericentral region. The induction of AKR1B8 appeared mostly in hepatocytes in the periportal region. The basal and inducible Ho-1 was located predominately in Kupffer cells. In the small intestine from WT mice, the inducible expression of Nqo1 and AKR1B8 appeared more obvious in the villus than that in the crypt.

**Specifications table**

**Table:** 

Subject area	Biology
More specific subject area	Pharmacology, Toxicology
Type of data	Image, text file, graph
How data was acquired	Images of immunohistochemistry were captured under a light microscope. Western immunoblot was scanned on an Odyssey scanner.
Data format	Raw, analyzed
Experimental factors	C57BL/6 Mice treated with sulforaphane (SFN)
Experimental features	Immunohistochemistry and western blot were used to analyze the expression of Nrf2 regulated Phase II enzymes in WT and *Nrf2*^*-/-*^ mice.
Data source location	The School of Medicine, Zhejiang University, Hangzhou, China
Data accessibility	The data are supplied with this article

**Value of the data**

• The data contain information for the generation and characterization of antibodies against Keap1, NQO1, AKR1B10 and HO-1.
• The data provide a histological view of inducible Nqo1, AKR1B8 and Ho-1 by SFN *in vivo*.
• The data may provide a better understanding on the chemopreventive effects of SFN.

## 1 Data

This dataset firstly shows the sensitivity and specificity of antibodies against Keap1, NQO1, AKR1B10 and HO-1. The antibodies were subsequently used for the immunohistochemical analysis on tissues sections from WT and *Nrf2*^*-/-*^ mice treated by SFN. Fig. 7, Fig. 8 display a similar inducible expression pattern of the detoxification enzymes by SFN, in comparison to that by BHA [1]. We evaluated the distribution of Nrf2 and/or its target genes in liver and small intestine in different cell types.

## 2 Experimental design, materials and methods

### 2.1 Chemicals and reagents

BHA and DL-Sulforaphane (SFN) were from Sigma-Aldrich Co., Ltd. (Shanghai, China). The antibody Gstα1/2 antiserum was kindly provided by Professor John Hayes (University of Dundee, Scotland).

### 2.2 Characterization of antibody

Antiserum against mouse Keap1 was raised in New Zealand White female rabbits against a purified full-length N-terminally His-tagged recombinant Keap1 that had been expressed from a pET-15b plasmid (Clontech, Mountain View, CA) in Escherichia coli Rosetta cells (Novagen, Merck, Darmstadt, Germany). The characterization of Keap1 antibodies were analyzed by Western immunoblotting (Fig. 1). The Keap1 antibody can react with the endogenous Keap1 and the exogenous GFP-Keap1.

**Fig. 2:**
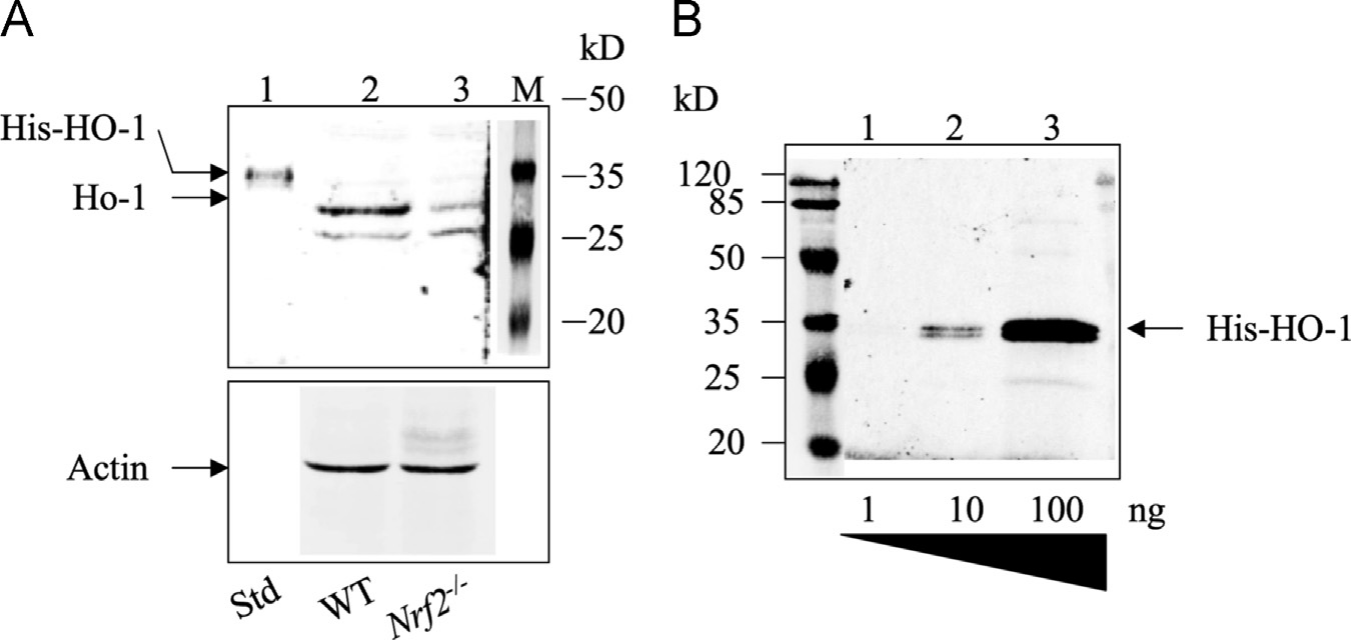
Characterization of antibody against Ho-1. (A) Cell extracts (100 μg) from the small intestine of WT (lane 2) and *Nrf2*^-/-^ (lane 3) mice were analyzed by Western immunoblotting with antibody against Ho-1. The Ho-1 antibody showed a decrease of Ho-1 in the small intestine from *Nrf2*^-/-^ mice. Lane 1, Purified His-tagged HO-1. (B) Purified His-tagged HO-1 (1–100 ng) analyzed with the Ho-1 antibody. The antibody reacted with 10 ng His-HO-1 (lane 2). The dilution of the antibody was 1:2000.

**Fig. 3:**
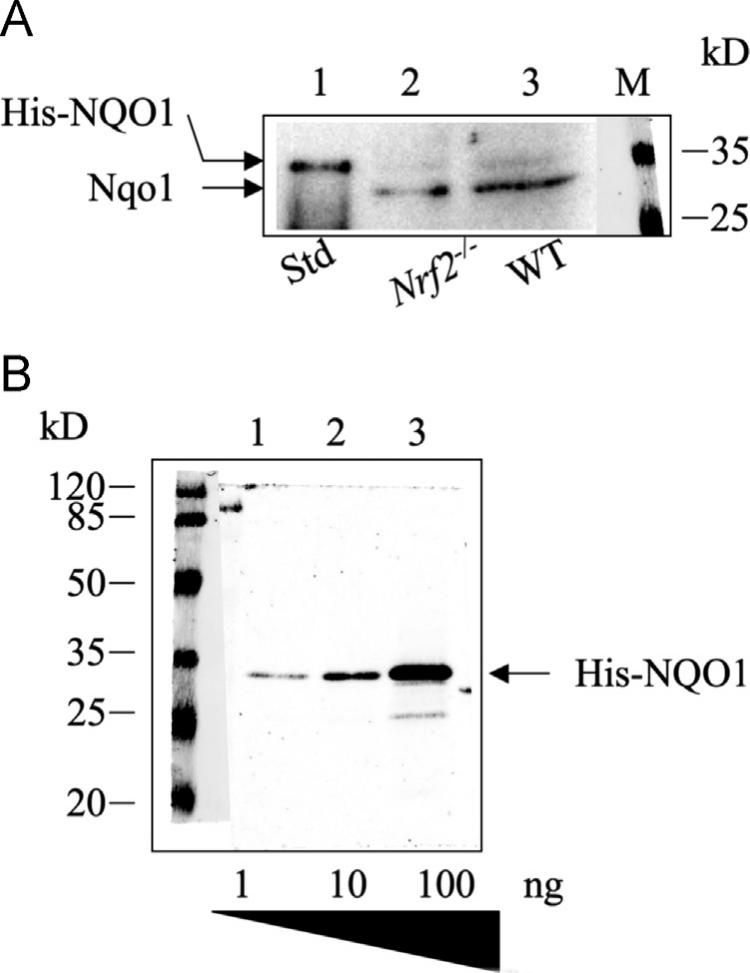
Characterization of antibody against NQO1. (A) Cell extracts (100 μg) from the small intestine of *Nrf2*^-/-^ mice (lane 2) and WT (lane 3) were analyzed by Western immunoblotting with antibody against NQO-1, which showed a decrease in the small intestine of *Nrf2*^-/-^ mice. Lane 1, Purified His-tagged NQO-1. (B) Purified His-tagged NQO-1 (1–100 ng) was analyzed with the NQO-1 antibody. The antibody reacted with 1 ng His-NQO-1. The dilution of the antibody was 1:3000.

**Fig. 4:**
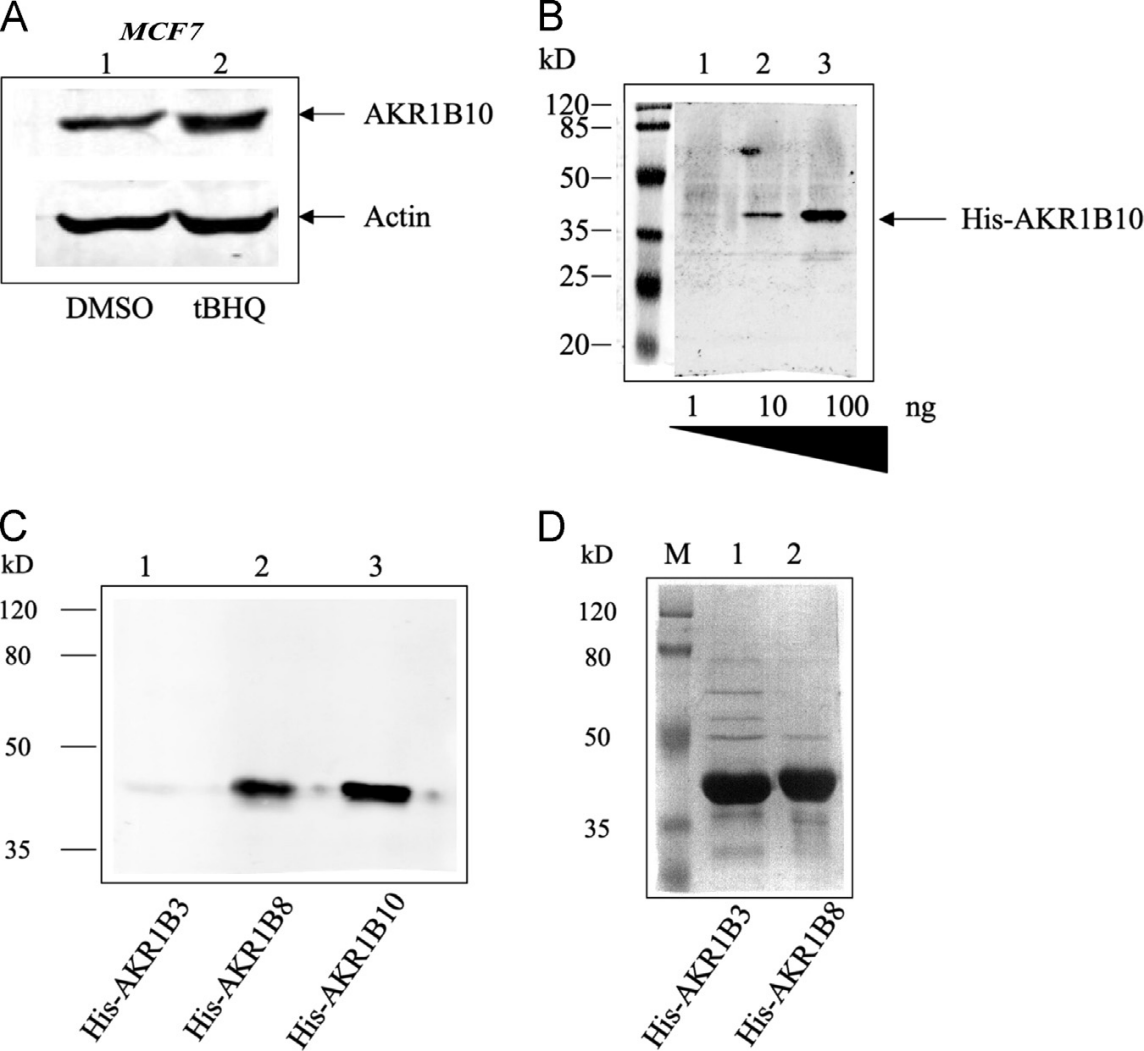
Characterization of antibody against AKR1B10. (A) Cell extracts (100 μg) from MCF7 cells treated with DMSO (lane 1) and 20 μM tBHQ (lane 2) were analyzed by Western immunoblotting with antibody against AKR1B10. AKR1B10 antibody detected the induction of AKR1B10 by tBHQ in MCF7 cells. (B) Purified His-tagged AKR1B10 (1–100 ng) was analyzed with the AKR1B10 antibody. The antibody reacted with 10 ng His-AKR1B10. The dilution of the antibody was 1:10000. (C) AKR1B10 reacts strongly with recombinant AKR1B10 and AKR1B8 but not AKR1B3. Full-length N-terminally His-tagged recombinant AKR1B3 (NM_009658.3), AKR1B8 (NM_008012), and AKR1B10, which had been expressed from a pETDuet-1 plasmid in *E*. *coli* Rosetta cells, were purified. The recombinant proteins (60 ng) were analysed by immunoblotting with AKR1B10 antibody. Coomassie stain of His-AKR1B3 (line 1) and His-AKR1B8 (lane 2) (10 μg) are shown in (D).

Antisera against HO-1, NQO1, and AKR1B10 were also raised in the rabbits against purified full-length N-terminally His-tagged recombinant human HO-1 (NM_002133.2), NQO1 (NM_000903.2), and AKR1B10 (NM_020299.4), which had been expressed from a pETDuet-1 plasmid (Novagen) in *E. coli* Rosetta cells. All antisera were affinity purified using Protein A resins and kits from Pierce Protein Biology Products (Thermo Scientific, USA) before use for Western immunoblotting and immunohistochemistry. The antisera against HO-1 and NQO1 react with mouse Ho-1 and Nqo1, respectively. The antiserum against AKR1B10 reacts with AKR1B8. The characterization of these antibodies is shown in Fig. 2, Fig. 3, Fig. 4.

### 2.3 Animal procedures

C57BL/6 wild-type (WT) mice were purchased from Shanghai Laboratory Animal Center (CAS, Shanghai, China). *Nrf2*^*-/-*^ mice were kindly provided by Prof. Masayuki Yamamoto (University of Tsukuba, Japan)[5]. Six–week-old male WT and *Nrf2*^*-/-*^ mice were divided into two groups (*n*=3), and given corn oil, or 25 mg/kg SFN orally once to induce Nrf2 regulated Phase II enzymes. Livers and small intestines were collected at 24 h or 48 h post SFN administration. Six–week-old male WT and *Nrf2*^*-/-*^ mice were divided into two groups (*n*=6), and given corn oil, or BHA (200 mg/Kg BW) by oral gavage daily for three days. The BHA was dissolved in corn oil. The group receiving oil was used as a negative control. Mice were sacrificed 4 h after the last BHA treatment. All animal procedures were performed with the approval of the Laboratory Animals Ethics Committee of Zhejiang University.

### 2.4 Western blot and immunohistochemistry

For Western blot, liver and small intestine extracts were prepared as previously reported [3], [4]. Protein samples were separated on SDS-PAGE gels and immunoblotting was carried out using the standard protocol. Immunoblotting with antibody against actin was performed to confirm equal loading for whole-cell extracts.

**Fig. 5:**
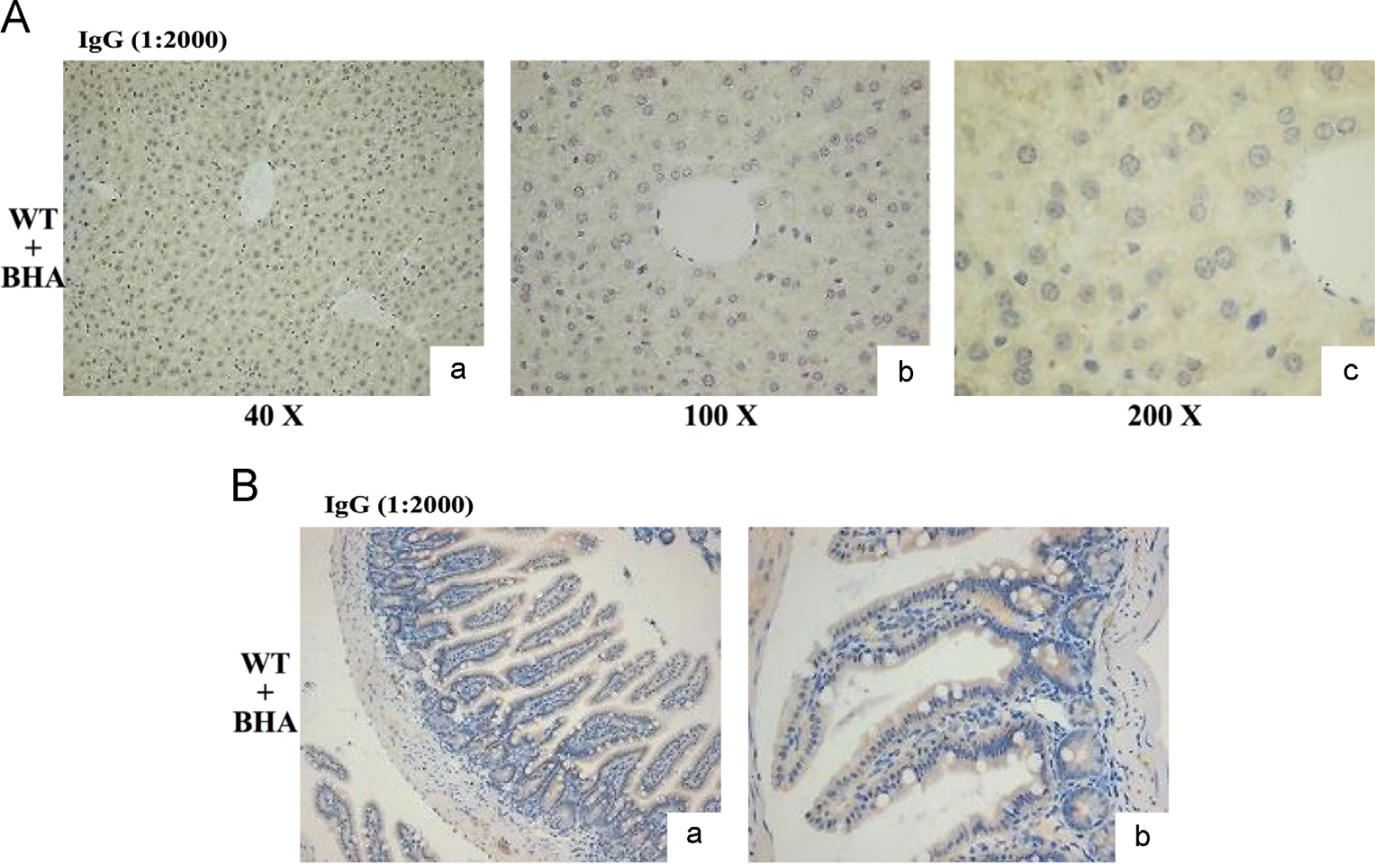
Immunohistochemical analysis of IgG in the liver (A) and small intestine (B) from BHA-treated mice. Sections of the liver and small intestine of WT mice given BHA (200 mg/kg) i.g. for 3 days were probed with IgG (1:2000 dilution). (a) Original magnification×40; (b) Original magnification×100; (c) Original magnification×200.

**Fig. 6:**
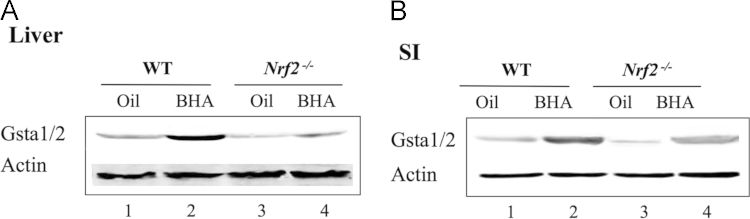
BHA increases the expression of Gstα1/2 in the liver and small intestine from WT mice. WT and Nrf2^-/-^ mice were given BHA (200 mg/kg) or oil (vehicle) by oral gavage for 3 days. Soluble extracts from the liver (A) and small intestine (SI) (B) were analyzed by Western immunoblotting with antibodies against Gstα1/2. Each lane shows the results for a sample from a single mouse. Actin was used as a loading control.

**Fig. 7:**
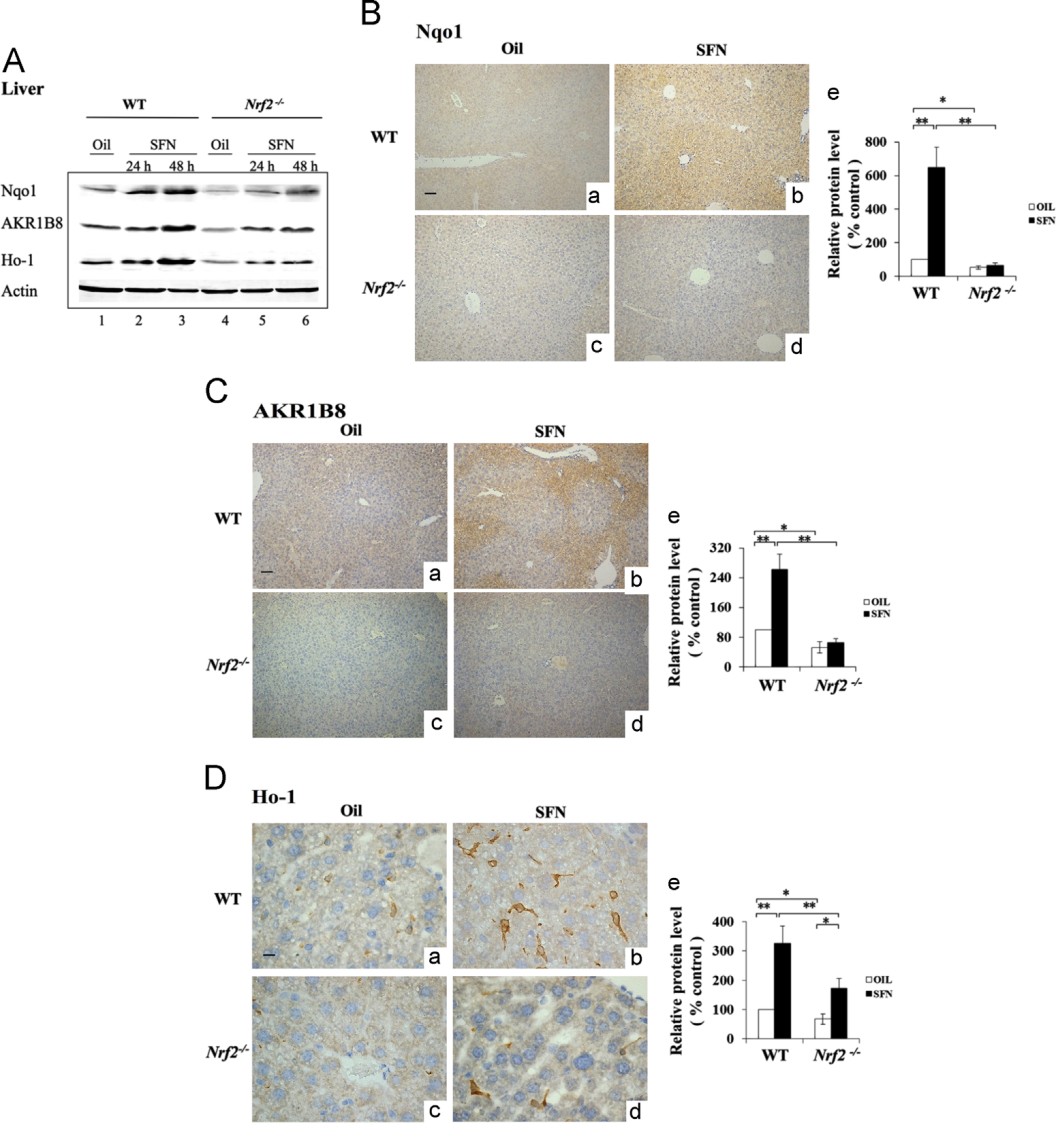
SFN increased the expression of Nqo1, AKR1B8 and Ho-1 in WT mouse liver. WT and *Nrf2*^*-/-*^ mice treated with oil or SFN (25 mg/kg) by single oral gavage. Mice were sacrificed 24 h and 48 h later (*n*=3). (A) Crude extracts from the liver were analyzed by Western immunoblotting with antibodies against Nqo1, Ho-1 or AKR1B8. Each lane shows the results for a sample from a single mouse. Actin was used as a loading control. Lane 1 and 4 show the results from the mice 48 h after the oil treatment. Sections of livers from mice 48 h after SFN (b and d) or oil treatment (a and c) were probed with antibodies against Nqo1 (B), AKR1B8 (C) or Ho-1 (D). (e) Semi-quantitative result of IHC (a–d). (B) and (C), Original magnification×40. (D), Original magnification×400. Scale bars, 50 μm; The control (oil) was set at 100%. Values are mean±SD. (*n*=3; *p<0.05, **p<0.01).

**Fig. 8:**
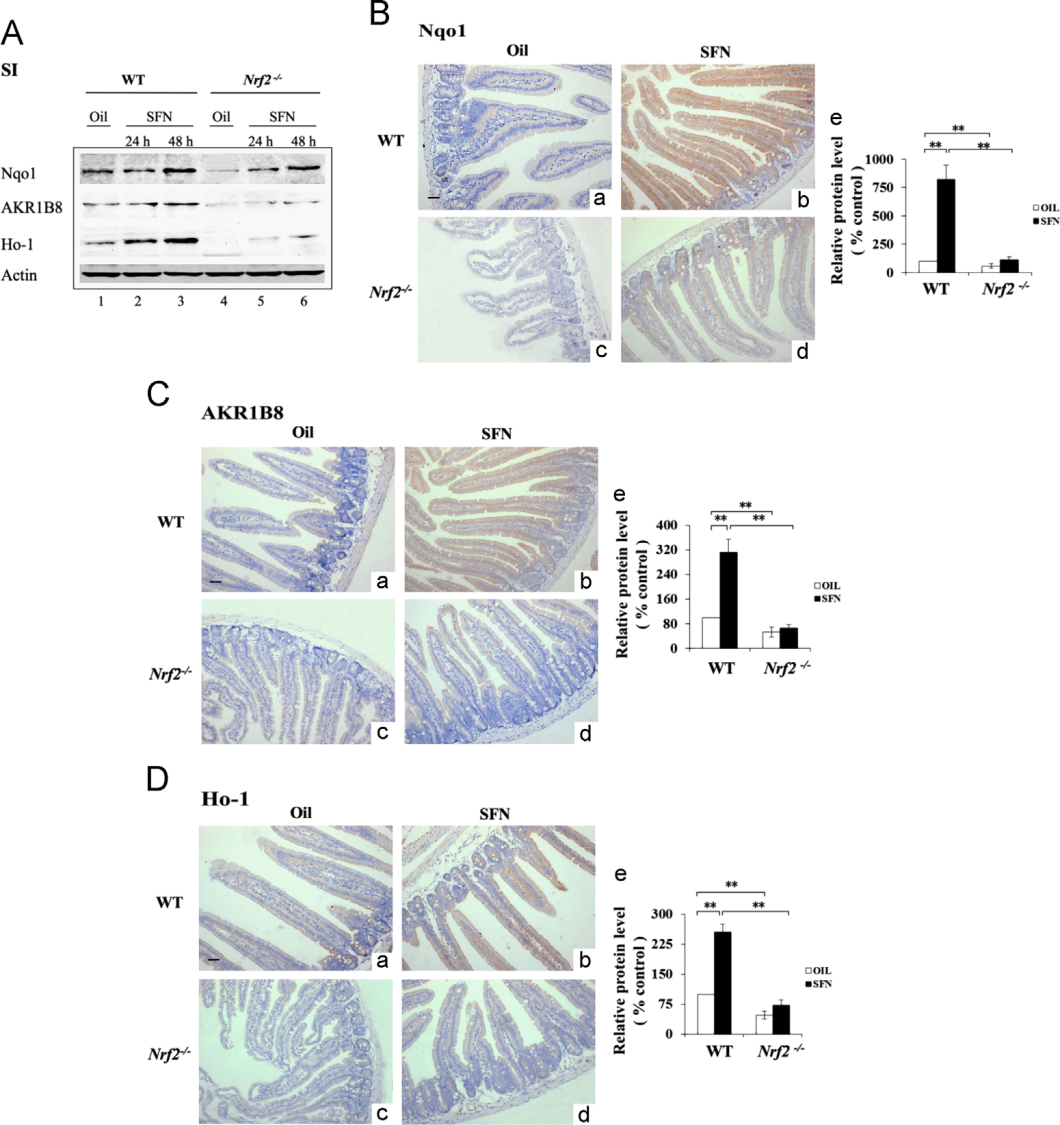
SFN increased the expression of Nqo1, AKR1B8 and Ho-1 in WT mouse small intestine. WT and *Nrf2*^*-/-*^ mice treated with oil or SFN (25 mg/kg) by single oral gavage. Mice were sacrificed 24 h and 48 h later (*n*=3). (A) Crude extracts from the small intestine were analyzed by Western immunoblotting with antibodies against Nqo1, Ho-1 or AKR1B8. Each lane shows the results for a sample from a single mouse. Actin was used as a loading control. Lane 1 and 4 show the results from the mice 48 h after the oil treatment. Sections of small intestine from the mice 48 h after SFN (b and d) or oil treatment (a and c) were probed with antibodies against Nqo1 (B), AKR1B8 (C) and Ho-1 (D). (e) Semi-quantitative result of IHC (a–d). (B–D), Original magnification×40. Scale bars, 50 μm; The control (oil) was set at 100%. Values are mean±SD. (*n*=3; *p<0.05, **p<0.01).

For immunohistochemistry, the livers and small intestines were fixed in 4% paraformaldehyde and embedded in paraffin [2], [6]. For immunohistochemistry, the antibodies against Ho-1, Nqo1, and AKR1B8 were used. The reacted antibody was visualized using Vector Laboratories ImmPRESS Detection kit, that employs a second antibody conjugated with horseradish peroxidase and a diaminobenzidine-based stain. All sections were counterstained with Mayer’s hematoxylin. The semi- quantitative result of IHC was based on the averaged value from three mice per group. For each mouse, three separate slides were analyzed. Images were captured under a light microscope (Olympus BX41, Shanghai, China) at 100×magnification. Image Pro Plus 6.0 (Media Cybernetics, Inc. ) was used to calculate the staining intensity. Five microscopic fields in tissues at 100×magnification were randomly selected and the integral optical density (IOD) of Nqo1, AKR1B8, and Ho-1 was calculated, and this was considered as the expression level. The control (oil) was set at 100%. As a negative control, sections of formalin-fixed liver and small intestine from the WT mice treated with BHA were probed with IgG, and no positive staining was observed ( Fig. 5, Fig. 6, Fig. 7, Fig. 8).

## Funding sources

Financial support for this study was provided by the National Natural Science Foundation of China (31170743, 81172230, 31370772, J1103603 and 31470752), the Zhejiang Natural Science Foundation (LZ12H16001), and Science and Technology Department of Zhejiang Province, China (2010C33156).

## Figures and Tables

**Fig. 1:**
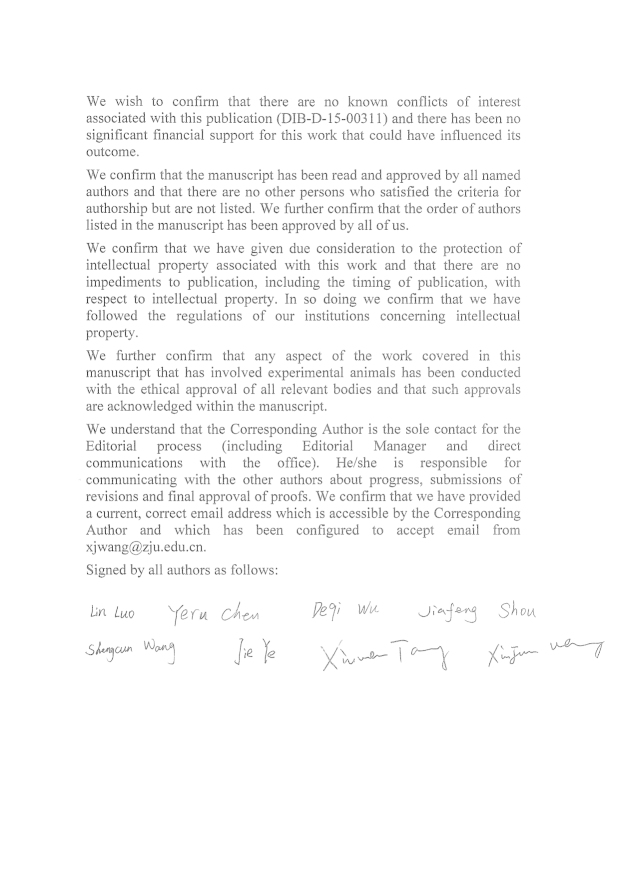


**Fig. 1:**
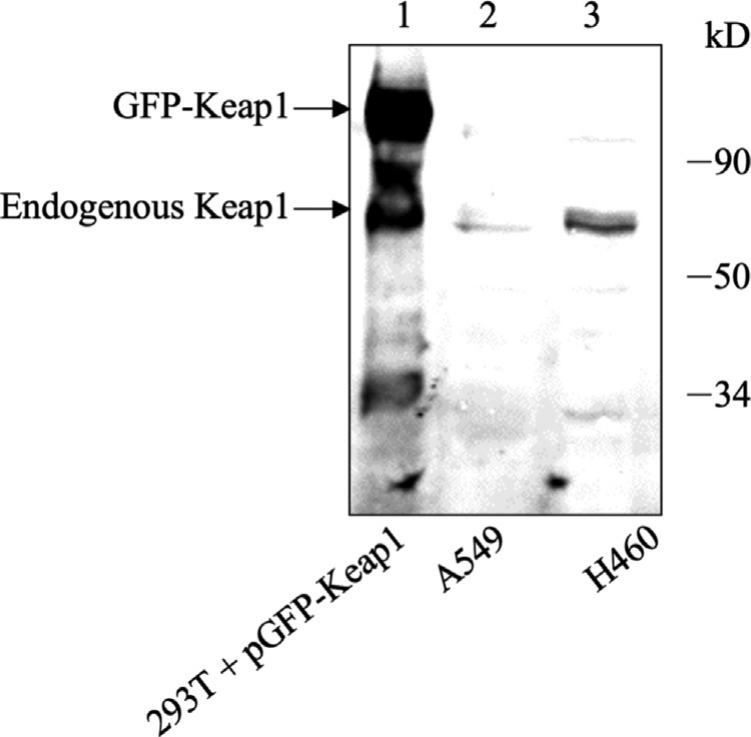
Characterization of antibody against Keap1. Cell extracts (30 μg) from Hek293T cells transfected with the mKeap1 expression plasmid pEGFP-mKeap1 (lane 1), A549 cells (lane 2), and H460 cells (lane 3) were analyzed by Western immunoblotting with antibody against Keap1 (1:1000 dilution). The Keap1 antibody reacted with the endogenous Keap1 and the exogenous GFP-Keap1.
